# Fishers’ knowledge about fish trophic interactions in the southeastern Brazilian coast

**DOI:** 10.1186/s13002-015-0012-8

**Published:** 2015-03-05

**Authors:** Milena Ramires, Mariana Clauzet, Walter Barrella, Matheus M Rotundo, Renato AM Silvano, Alpina Begossi

**Affiliations:** Laboratório de Ecologia Humana, Programa de Pós Graduação em Sustentabilidade de Ecossistemas Costeiros e Marinhos (ECOMAR), Universidade Santa Cecília – UNISANTA, Rua Cesário Mota, 08. Bloco F, Santos, SP CEP: 11045-040 Brazil; Fisheries and Food Institute – FIFO/UNISANTA, Rua Cesário Motta, 08. Sala 82 F, Santos, SP CEP:11045-040 Brazil; Acervo Zoológico da Universidade Santa Cecília (AZUSC), Rua Oswaldo Cruz, 266, Santos, SP CEP: 11045-907 Brazil; Departamento de Ecologia, Universidade Federal do Rio Grande do Sul, CP 15007, CEP: 91501-970 Porto Alegre, RS Brazil; CAPESCA/NEPA/UNICAMP, Rua Albert Einstein 291, Campinas, SP CEP 13083-852 Brazil

**Keywords:** Ethnoecology, Artisanal fisheries, Fish ecology, Fisheries management, Ethnoichthyology, Ilhabela

## Abstract

**Background:**

Data derived from studies of fishers’ local ecological knowledge (LEK) can be invaluable to the proposal of new studies and more appropriate management strategies. This study analyzed the fisher’s LEK about trophic relationships of fishes in the southeastern Brazilian coast, comparing fishers’ LEK with scientific knowledge to provide new hypotheses.

**Methods:**

The initial contacts with fishers were made through informal visits in their residences, to explain the research goals, meet fishers and their families, check the number of resident fishers and ask for fishers’ consent to participate in the research. After this initial contact, fishers were selected to be included in the interviews through the technique of snowball sampling. The fishers indicated by others who attended the criteria to be included in the research were interviewed by using a semi-structured standard questionnaire.

**Results:**

There were interviewed 26 artisanal fishers from three communities of the Ilhabela: Jabaquara, Fome and Serraria. The interviewed fishers showed a detailed knowledge about the trophic interactions of the studied coastal fishes, as fishers mentioned 17 food items for these fishes and six fish and three mammals as fish predators. The most mentioned food items were small fish, shrimps and crabs, while the most mentioned predators were large reef fishes. Fishers also mentioned some predators, such as sea otters, that have not been reported by the biological literature and are poorly known.

**Conclusions:**

The LEK of the studied fishers showed a high degree of concordance with the scientific literature regarding fish diet. This study evidenced the value of fishers’ LEK to improve fisheries research and management, as well as the needy to increase the collaboration among managers, biologists and fishers.

## Background

The number of studies on ethnoecology, which addresses the local knowledge and interactions between human populations and natural resources, have increased considerably along the last two decades [1-9]. This knowledge of local populations has been regarded in previous studies as being either indigenous knowledge, local ecological knowledge (LEK) or traditional ecological knowledge (TEK) [10-12]. The ethnoecology as a research discipline addresses the cultural expression of a human population or community about their comprehension of the biological world as well as on the food chain. This comprehension can be regarded as an intellectual tradition, which can be transmitted among people and between generations [13], interpreting and applying information about relationships in the natural world [14]. According to Marques [12], the ethnoecology includes the study of the knowledge, beliefs, behaviors and feelings that influence all interactions between people and ecosystems, including use of natural resources and impacts.

Considering fishing resources, fishers have been providing important information on the biology and ecology of fish, even about aspects on which scientific knowledge is lacking, such as reproduction, migration and trophic interactions [15-18]. Many tropical developing countries lack biological and fisheries data about fishing resources, which may be an obstacle to devise effective fisheries management plans [19]. Therefore, fishers’ LEK have been an invaluable and useful source of new data about fish ecology, migration, reproduction, feeding habits, temporal patterns in the abundance of exploited stocks, extinction risk and environmental impacts worldwide, such as the studies about fishers’ LEK in the southeast Asia [20]), south Pacific [2,21], Artic [4,7] and in an African estuary [8].

According to Alves and Souto [22] the field of ethnoichthyology is particularly prominent in Brazil, representing 12.3% of published studies on the discipline of Among these studies on LEK about fish trophic interactions, Costa Neto and Marques [23] described the perception of fishermen from northeastern Brazilian coast on behavior (sound production), playback and trophic ecology of fish; Souza and Barrella [24,25] analyzed the knowledge of traditional fishermen from southeastern Brazil (Ecological Station of Juréia Itatins) in relation to habitat, trophic ecology and spatial distribution of fish; Silvano and Begossi [26] analyzed the LEK fishermen on fish behavior and biology in the Piracicaba River; Fernandes-Pinto and Marques [27] studied the cognitive models of fishermen from Guaraqueçaba (PR), with emphasis on knowledge about fish; Begossi and Silvano [28] analyzed the diet of dusky grouper in the Brazilian coast by using conventional scientific methods associated with fishers’ LEK and Silvano and Begossi [29] adopted the same approach to study the diet of bluefish. Begossi *et al.* [30] show that fishers’ LEK is more detailed about target fish species, illustrating that with the snappers (Lutjanidae), which have been fished by artisanal fishers in the southeastern and northeastern Brazilian coasts. Some of these species of snappers, such as *Lutjanus analis* and *L. synagris*, may had been adversely affected by the fishing pressure, but local fishers could collaborate in management proposals and they have important knowledge, which can fill gaps in the current scientific knowledge about these fishes and support future management initiatives [30].

These studies have shown that Brazilian fishers have a detailed knowledge about fish trophic interactions. Therefore, fishers’ LEK can provides rapid and reliable approaches to bring information on diet and trophic relationships of several other commercially important species of fish, most of which were not studied the Brazilian coast.

Both the LEK and the academic ecological knowledge are based on systematic and empirical observations of nature [14,31]. Some studies have emphasized the potential application of LEK as an input of information to subsidize and improve resource’s management [32,33]. Therefore, data derived from studies of LEK can be invaluable to the proposal of new scientific hypotheses, new studies and more appropriate management strategies [1,7,15]. For example, Silvano & Valbo-Jorgensen [15] make a review of scientific publications on fishers’ LEK from Brazil (coastal and freshwater) and Southeast Asia, comparing data from LEK with information from the scientific literature to elaborate 29 hypotheses on fish ecology, biology and behavior, thus indicating the potential of LEK to contribute with fisheries management.

Begossi [34] indicates four important factors to promote the integrated management of fishing resources: (1) understanding the natural environment on which fishing occurs, besides the use of natural resources by the local population; (2) knowledge about the marine area used by fishers, such as the location of fishing grounds of each target species; (3) understanding fishers’ behavior, such as their choices and decisions; and (4) the detailed fishers’ LEK about fish.

In Brazil and in other tropical countries, coastal artisanal fisheries exploit several fish species [30,35], some of these fish may be overexploited, such as some reef fishes [28,36], and there is a shortage of biological knowledge about many fish species to inform management decisions. In this sense, this study aims to record, describe and analyze the fisher’s LEK about trophic relationships of 24 fish species in an island of the southeastern Brazilian coast. This study also compares fishers’ LEK with existing scientific knowledge, to provide new hypotheses and information about fish trophic ecology, which could contribute to management and conservation of coastal fishes and fishing resources.

## Methods

This research was conducted with three communities of artisanal fishers, who are locally called *caiçaras*, in three beaches of the island of Ilhabela, in the southeastern Brazilian coast: beaches of Jabaquara, Fome and Serraria. Although they may perform some other economic activities, these fishers dedicate mainly to artisanal small-scale fisheries, fishing in a daily basis to acquire food, animal protein and monetary resources from fish sales. The LEK of these fishers was not yet recorded to shows its potential contributions to fisheries management. At the time of the study, there were five families living in the community of Jabaquara, eight in Fome and 16 in Serraria [37]. This survey was authorized by the Ethics Committee for Research Involving Human Beings of the University of Santa Cecília (register CEP/ UNISANTA N° 10/07).

The initial contacts with fishers were made through informal visits in their residences, to explain the research goals, meet fishers and their families, check the number of resident fishers and ask for fishers’ consent to participate in the research. After this initial contact, the communities were revisited in the winter and summer periods between the years 2005 and 2007, where the fishers were selected to be included in the interviews through the technique of snowball sampling [38]. Following this technique, the sampling started by interviewing a fisher considered by the rest of community as being experienced; after the interview, this fisher indicated other fishers who might be interviewed according to defined criteria: older than 18 years, and living and fishing in the studied community for at least 10 years. After each interview, the interviewed fisher was again asked to indicate other fishers who filled the above criteria.

The fishers indicated by others and whom attended the criteria to be included in the research were interviewed by using a semi-structured standard questionnaire (more details on this methodology are in Ramires *et al*. [37]). We were able to interview fishers from all resident families of the studied communities. These interviews included photographs of 24 fish species that are often caught by commercial fishers of the southeastern Brazilian coast, to allow fishers name and recognize each fish being studied. These fish species were previously selected to be included in this study, as described in more detail in a previous and related research [39]. The photos of the studied fishes were shown to fishers always in the same order, which was randomly defined by raffle, following methods adopted in previous ethnoichthyological surveys [3,40-42]. After viewing the photo and naming the fish species, fishers were asked about the fish feeding habits and its potential predators, to allow a more detailed analysis of trophic relationships, as in a previous survey [42]. The consensus among interviewed fishers was achieved through the quantitative analysis of the proportion of citations: those answers or aspects that were more often mentioned by the interviewed fishers were also considered as more relevant LEK data [40,42,43]. The information gathered from fishers’ LEK was compared with information from the scientific literature, following the approach of tables of compared cognition [44], which are useful to readily compare these two knowledge sources. The literature data were gathered through a comprehensive review of published studies about the 24 studied fish species.

The results described in the tables of compared cognition were analyzed following the conceptual framework proposed by Silvano & Valbo-Jorgensen [15], who integrate data from both kinds of knowledge (LEK and scientific), to formulate new and testable hypotheses, which could ultimately improve fisheries research and management measures. This framework indicates a relative measure of the degree to which a hypothesis formulated from fishers’ LEK agrees with information from the scientific literature, thus providing an objective and systematic way of analyzing the potential of fishers’ LEK to complement conventional scientific research [15]. This measure is denominate likelihood and can be: high, when quotations from fishers’ LEK agree well with the scientific literature; medium, when these two kinds of knowledge cannot be properly compared due to lack of scientific information, and only fishers’ LEK is available; and low, when hypotheses devised from fishers’ LEK are unexpected or even contradicts the existing biological data [15].

### Data analysis

In order to check the most cited food items for the 24 fish species studied, a Kruskall–Wallis test was made comparing the median percent of fishers who cited each food item, considering each species as a replicate in the analysis. This same analysis was made to check the most cited predators for the 24 fish species studied.

## Results

**Figure 1:**
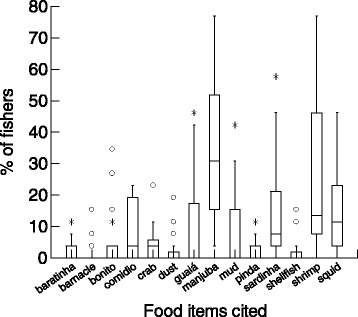
**Comparison of median (lines inside boxes) values of the percent of interviewed fishers (n = 26) who cited each food item for 24 coastal fish species, in Ilhabela island, southeastern Brazilian coast.** Identifications of food items and the percent of citations for each fish species are in Table 1. This comparison considers only those food items cited by more than 10% of the interviewed fishers, for three or more fish species.

There were interviewed 26 artisanal fishers from the three studied communities, one fisher from each family. These fishers showed a detailed knowledge about fish diet and cited 17 distinct food items consumed by the studied fishes, ranging from only two items eaten by the bonito (*Euthynnus alleteratus*) to eight items consumed by the tiniuna *(Abudefduf saxatilis*) (Table 1). Overall, fishers’ LEK about fish diet showed a high agreement with information from the scientific literature, for most of the studied species (Table 1). Considering the median percent of fishers who cited each food item among all fish species, some items were more cited (Kruskall–Wallis H = 124.7, df = 13, p > 0.001), such as sardinha, manjuba, shrimp and squid (Figure 1).

**Table 1:** Comparison between fishers’ LEK* and the scientific literature about the diet of fish species in Ilhabela (Brazil)**

**Fish species (Local name)**	**Food items from fishers’ LEK**	**Food items from the scientific literature**	**Likelihood**
*Bodianus rufus* (Godião)	Mud (42), Guaiá^a^ (35), Manjuba (35), Shrimp (19), Shellfish (15), Comidio^b^ (12)	Shellfish, crabs, equinoids, may be cleaners of larger fish [45-49]	High
*Epinephelus marginatus* (Garoupa)	Sardinha (46) Guaiá (38) Bonito (35) Manjuba (27) Crab (23) Rotten bait (12)	Crabs, moluscs (mainly cephalopods) and fish (mainly planctivorous) [47,50-55]	High
*Epinephelus morio* (Garoupa)	Sardinha (46) Guaiá (42) Bonito (35) Manjuba (31) Crab (23)	Fish and invertebrates, mainly crustaceans and cephalopods [51,52,56]	High
*Caranx latus* (Xaréu)	Manjuba (77) Squid (23) Comidio (19) Shrimp (12)	Mainly fish and, in lower proportion, shrimp and other invertebrates, including pteropods and copepods [47,51,57,58]	High
*Umbrina coroides* (Betara)	Shrimp (73) Squid (15) Mud (12) Manjuba (12)	Benthic organisms, mainly crustaceans (Amphipoda and Mysidacea) [59-61]	High
*Mycteroperca bonaci* (Badejo)	Shrimp (35) Manjuba (35) Sardinha (23) Squid (19) Guaiá (15)	Adults eat mainly fish and juveniles eat crustaceans and also cephalopods [46,51,56]	High
*Mugil curema* (Parati)	Water’s foam (58) Sand (19) Mud (19) Dust (19)	Juveniles eat plankton and adults eat plant material, microalgae, benthic microorganisms, copepods, polichaeta and detritus from the substrate [45,62-64]	Medium
*Seriola lalandi* (Olhete)	Squid (46) Manjuba (42) Comidio (27) Sardinha (19)	Fish, cephalopods and crustaceans [65,66]	Medium
*Bodianus pulchellus* (Godião)	Guaiá (46) Mud (27) Shrimp (15) Barnacle (15) Shellfish (12) Pindá^c^ (12) Don’t know (12)	Crustaceans, mollusks and ectoparasites from other fish [45-47,49]	Medium
*Oligoplites saliens* (Guaivira)	Manjuba (73) Comidio (23) Shrimp (12) Squid (12)	Mainly fish and, in a lesser extent, cephalopods and crustaceans. This species may also eats scales from other fish [46,57]	High
*Pomatomus saltatrix* (Anchova)	Manjuba (69) Sardinha (35) Squid (27) Comidio (19) Shrimp (12)	Mainly fish, eventually mollusks, crustaceans and cephalopods [46,67-72]	High
*Caranx crysos* (Xarelete)	Manjuba (73) Comidio (23) Squid (19) Shrimp (12) Sardinha (12)	Fish, cephalopods, crustaceans and other benthic invertebrates [46,58,59]	High
*Micropogonias furnieri* (Corvina)	Shrimp (69) Squid (27) Manjuba (19)	Crustaceans, cephalopods, anelids and fish [46,52,59,61,68,73]	High
*Cynoscion jamaicensis* (Goete)	Shrimp (54) Squid (31) Manjuba (27) Sardinha (12) Comidio (12)	Mainly fish and crustaceans [61,74]	High
*Stegastes fuscus* (Café torrado)	Shrimp (35) Guaiá (31) Mud (27) Shellfish (15)	Mainly algae, crustaceans, cnidarians and small invertebrates [45,46,75,76]	High
*Scomberomorus brasiliensis* (Sororoca)	Manjuba (54) Comidio (19) Sardinha (19) Shrimp (12)	Mainly fish, but also cephalopods and crustaceans [46,68,77,78]	High
*Centropomus parallelus* (Robalo)	Shrimp (65) Manjuba (31) Squid (15)	Fish and crustaceans [46,79]	High
*Mycteroperca acutirostris* (Miracelo)	Shrimp (54) Manjuba (50) Comidio (23) Squid (15)	Mainly fish, plankton and small crustaceans [47,51,80]	High
*Abudefduf saxatilis* (Tiniuna)	Shrimp (31) Guaiá (19) Mud (19) Barnacle (15) Baratinha^d^ (12) Bonito (12) Manjuba (12) Dust (12)	Plankton, small invertebrates, crustaceans and algae [45,47,81]	High
*Euthynnus alleteratus* (Bonito)	Manjuba (77) Comidio (23)	Fish, crustaceans, heteropods and cephalopods [46,77,78]	Medium
*Trichiurus lepturus* (Espada)	Sardinha (54) Manjuba (46) Squid (23) Comidio (15)	Fish, cephalpods and crustaceans [77,82,83]	High
*Mugil liza* (Tainha)	Water’s foam (62) Mud (31) Sand (12) Manjuba (12)	Plankton and organic detritus [45,63]	High
*Menticirrhus americanus* (Betara)	Shrimp (77) Manjuba (23) Squid (12) Crab (12)	Benthic organisms, mainly polichaeta and crustaceans [46,59,61]	Medium
*Lutjanus synagris* (Vermelho)	Shrimp (38) Squid (35) Sardinha (31) Manjuba (23) Bonito (15)	Prefers to eat crustaceans and fish, but eats also equinoderms, polichaeta, gastropods and cephalopods [52,59,68,84]	High

*n = 26 interviewed fishers

**showing the degree of likelihood or agreement between these two kinds of knowledge according to Silvano and Valbo-Jorgensen [15] and the values in parenthesis are the percent of interviewed fishers who mentioned each food item, showing only those items mentioned by more than 10% of the interviewed fishers.

^a^Guaiá = small crab that lives among rocks, see Silvano and Begossi [42] for more details.

^b^Comidio = large schools formed by several species of small fish.

^c^Pindá = black sea urchin (*Echinometra lucunter*).

^d^Baratinha = crustacean (*Ligia exotica*).

The values in parenthesis are the percent of interviewed fishers who mentioned each food item, showing only those items mentioned by more than 10% of the interviewed fishers. Fish species are shown in the same order that they were shown to the interviewed fishers.

Besides their knowledge about fish diet, the studied fishers showed also a detailed knowledge about trophic relationships between fish and other marine organisms: they cited six fishes and three mammals as potential predators of the studied fishes (Table 2). Among the most cited predators are two aquatic mammals, the dolphin and the sea otter, which, according to fishers, prey on respectively 15 and 7 of the studied fish species (Table 2). In both cases, fishers possibly acquired this knowledge from environmental observations of the feeding behavior of these aquatic mammals. For example, the interviewed fishers mentioned that, during their fishing activities, they observe the dolphin following fish schools to prey on them. The interviewed fishers also mentioned that they observe the sea otter catching fish along the rocky shores.

**Table 2:** Comparison between fishers’ LEK* and the scientific literature about the predators of fish species in Ilhabela (Brazil)**

**Fish species (Local name)**	**Predators from fishers’ LEK**	**Predators from the scientific literature**	**Likelihood**
*Bodianus rufus* (Godião)	Shark (12)	Lutjanidae - Teleostei [85]	Low
*Epinephelus marginatus* (*Garoupa*)	Caranha^a^ (27) Mero^b^ (23) Shark (15) Otter (15)	No data	Medium
*Epinephelus morio* (Garoupa)	Caranha (23) Mero (23) Shark (15) Otter (15)	No data	Medium
*Caranx latus Xaréu*	Dolphin (42) Caranha (23) Espada (23) Mero (23) Shark (12) Porpoise (12)	Coryphaenidae – Teleostei [86]; Laridae – sea birds [87]	Low
*Umbrina coroides* (Betara)	Shark (27) Caranha (19) Mero (19) Espada (15) Dolphin (12) Garoupa (12)	No data	Medium
*Mycteroperca bonaci* (Badejo)	Caranha (23) Mero (23) Garoupa (12) Otter (12)	No data	Medium
*Mugil curema* (Parati)	Dolphin (58) Espada (23) Mero (23) Caranha (19) Shark (12) Otter (12)	Centropomidae - Teleostei [46]; Sulidae – sea bird [88]; Delphinidae – dolphin [89]	High
*Seriola lalandi* (Olhete)	Dolphin (38) Mero (19) Caranha (15) Shark (12) Otter (12)	Coryphaenidae - Teleostei [88]	Low
*Bodianus pulchellus* (Godião)	Caranha (23) Mero (23) Garoupa (19) Shark (12)	No data	Medium
*Oligoplites saliens* (Guaivira)	Dolphin (19) Shark (15) Caranha (15) Mero (15)	No data	Medium
*Pomatomus saltatrix* (Anchova)	Dolphin (62) Shark (23) Caranha (23) Mero (19) Garoupa (12) Otter (12)	Alopiidae, Carcharhinidae, Lamnidae, Squatinidae – Elasmobranchii [90-93]; Scombridae, Xiphiidae, Pomatomidae - Teleostei [94-96]	Low
*Caranx crysos* (Xarelete)	Dolphin (46) Shark (38) Caranha (19) Espada (15) Mero (15) Garoupa (12)	Scombridae, Coryphaenidae, Istiophoridae, Sphyraenidae – Teleostei [85,86,97,98]; Laridae – sea bird [87]	Low
*Micropogonias furnieri* (Corvina)	Dolphin (15) Caranha (15) Mero (15) Shark (12)	Triakidae - Elasmobranchii [99]; Sciaenidae – Teleostei [100,101]; Sulidae – sea bird [90]; Delphinidae – dolphin [102,103]	High
*Cynoscion jamaicensis* (Goete)	Caranha (23) Shark (19) Espada (19) Mero (19) Dolphin (12)	Delphinidae – dolphin [102,104]	Medium
*Stegastes fuscus* (Café torrado)	Garoupa (31) Mero (27) Caranha (23)	Serranidae – Teleostei [85]	High
*Scomberomorus brasiliensis* (Sororoca)	Dolphin (46) Caranha (19) Espada (19) Mero (19) Shark (15)	No data	Medium
*Centropomus parallelus* (Robalo)	Mero (38) Caranha (19) Shark (12)	No data	Medium
*Mycteroperca acutirostris* (Miracelo)	Mero (35) Caranha (27) Otter (19) Garoupa (15) Shark (12)	No data	Medium
*Abudefduf saxatilis* (Tiniuna)	Garoupa (35) Mero (31) Caranha (19)	Serranidae, Labridae, Rachycentridae – Teleostei [85,105,106]	High
*Euthynnus alleteratus* (Bonito)	Dolphin (54) Mero (27) Garoupa (23) Caranha (15) Shark (12)	Scombridae, Coryphaenidae, Istiophoridae, Xiphiidae – Teleostei [86,89,97,98,107-112]; Laridae – sea bird [87]	Low
*Trichiurus lepturus* (Espada)	Dolphin (50) Mero (23) Espada (19) Shark (12) Caranha (12)	Carcharhinidae, Lamnidae, Sphyrnidae - Elasmobranchii, [93,113-118]; Scombridae, Coryphaenidae, Istiophoridae, Sciaenidae, Trichiuridae, Rachycentridae, Pomatomidae – Teleostei [69,89,97,98,106,111,119]; Delphinidae, Pontoporiidae – dolphin [102-104,118,120,121]	High
*Mugil liza* (Tainha)	Dolphin (54) Shark (15) Caranha (15) Garoupa (15) Mero (15)	Pomatomidae – Teleostei [46]; Sulidae – sea bird [89]	Low
*Menticirrhus americanus* (Betara)	Shark (23) Dolphin (15) Caranha (15) Garoupa (12) Mero (12)	Carcharhinidae – Elasmobranchii [114,122]; Delphinidae – dolphin [118]	High
*Lutjanus synagris* (Vermelho)	Mero (31) Caranha (23) Garoupa (19) Dolphin (12) Espada (12)	Serranidae – Teleostei [85]; Delphinidae – dolphin [89]	High

^*^n = 26 interviewed fishers.

**showing the degree of likelihood or agreement between these two kinds of knowledge according to Silvano and Valbo-Jorgensen [15] and the values in parenthesis are the percent of interviewed fishers who mentioned each predator, showing only those predators mentioned by more than 10% of the interviewed fishers.

^a^Caranha is a large fish of the Lutjanidae (*Lutjanus* sp.).

^b^Mero is the goliath grouper (*Epinephelus itajara*).

The values in parenthesis are the percent of interviewed fishers who mentioned each predator, showing only those predators mentioned by more than 10% of the interviewed fishers. Fish species are shown in the same order that they were shown to the interviewed fishers.

**Figure 2:**
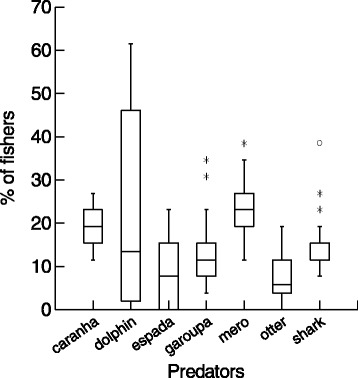
**Comparison of median (lines inside boxes) values of the percent of interviewed fishers (n = 26) who cited each predator for 24 coastal fish species, in Ilhabela island, southeastern Brazilian coast.** Identifications of predators and the percent of citations for each fish species are in Table 1. This comparison considers only those predators cited by more than 10% of the interviewed fishers, for three or more fish species.

Contrarily to the pattern observed for fish diet (Table 1), overall the agreement between fishers’ LEK and the scientific literature on fish predators was medium or low, as for some species we could not find scientific data to compare with LEK, while for others LEK and the scientific literature mentioned distinct predators (Table 2). For example, the sea otter was not mentioned and the dolphin was only occasionally mentioned as predator of the studied fishes by the scientific literature (Table 2). The available scientific studies usually mention large pelagic fish, sharks and sea birds as predators, while fishers mentioned mainly aquatic mammals, sharks and large reef fish, such as groupers and large lutjanids (Table 2). Indeed, considering the median percent of fishers who cited each predator among all fish species, the large reef fishes caranha and mero were more cited than other predators (Kruskall–Wallis H = 59.5, df = 6, p > 0.001, Figure 2).

## Discussion

This study followed the same quantitative analytical approach adopted in previous studies of fisher’s LEK, which also show that fishers have a detailed and accurate LEK about fish trophic interactions in the Brazilian coast [28,29,42,43,123]. A high agreement between fishers’ LEK and scientific information indicates that one source of information reinforces the other, and the information from fishers’ LEK is thus potentially applicable to improve fisheries management in Brazil [15,29,34,42,124]. Therefore, the usually high agreement between fishers’ LEK here reported about fish diet and the available scientific information (Table 1), could provide new and useful data for fish conservation and fisheries management purposes. For example, data from fishers’ LEK can be used to elaborate conceptual models of food webs, which can indicate the main trophic interactions among fish, their prey and their predators in coastal ecosystems, as has been made in studies in the Brazilian coast [42,44], and in an African estuary [8].

The LEK of the studied fishers in the Ilhabela Island showed a high degree of concordance with the scientific literature regarding fish diet, especially for commercial fishes. For example, fishers mentioned that the anchova (*Pomatomus saltatrix*) eats manjuba, sardinha, squid, small fishes and shrimp (Table 1). In a previous survey, Silvano & Begossi [29] compare fishers’ LEK about the diet of the anchova with stomach content analysis of this species in two sites of the southeastern Brazilian coast, observing a high agreement between these two data sources (LEK and biological sampling), which both indicate that this fish eats mainly fish and also shrimp and squid.

Most of the literature information about fish came from general publications with taxonomic purposes, which do not include stomach content analyses [45-47,51,71,78]. Relatively few of the consulted publications report detailed analyses of feeding behavior or diet (stomach contents) of the studied fishes [48,57,60,75,82]. Furthermore, some of these dietary surveys address just a few commercial species, such as corvina [73], garoupa [50,54] and anchova [67,69,70] and most of them were conducted in other regions of the world, not in the southeastern Brazilian coast. In such context, the detailed data on fish diet here provided from fishers’ LEK could help to fill this gap in the biological knowledge about coastal fishes of the southern Atlantic. For example, fishers mentioned five food items consumed by the goete (*Cynoscion jamaicensis*), while the literature only indicates that this fish eats fish and crustaceans [47].

Fishers acquire this detailed knowledge on fish diet through daily observation of stomach contents while they clean fish and find parts of food items, such as crabs, fish, shrimp, among other items. Fishers may also acquire knowledge on fish diet through the manipulation of food item as baits, as there is usually a coincidence between items mentioned as food for fish and used as bait [29,43]. This is an important point, since the citations of sardinha, manjuba, shrimp and squid might overemphasize the importance of these resources as food items, as fishers might be tempted to mention the common food they use as bait.

Considering that scientific biological studies are often limited in time frame or number of fish analyzed (sampling effort), fishers LEK could improve scientific research, by providing useful hypotheses to be investigated [15,44]. In Ilhabela Island fishers mentioned crabs, and more specifically a small rocky crab called guaiá as an important food item for several fish species, especially the reef fishes (Table 1, Figure 1). Other important food item for coastal fishes according to the interviewed fishers in Ilhabela Island are the pelagic and schooling small fishes sardinha (Clupeidae) and manjuba (Engraulidae) (Figure 1). Similarly, crabs and small fishes (clupeids and engraulids) have been also mentioned by fishers as being important food items for coastal fishes, including important commercial species, such as grouper and anchova, in other regions of the southeastern Brazilian coast [28,29,42,43], thus indicating a potentially relevant trophic link in coastal ecosystems. Fishers’ LEK about fish diet in the south Pacific has been useful to evidences potential negative effects of tuna bait fishery on reef fishes, as small fishes used as bait are important food items of large reef predators [1]. Our results can be useful to highlight these trophic relationships, which have not been studied in detail and may not seem obvious to fisheries’ managers.

Information about predators of coastal fishes is even scarcer in the biological literature than information about fish diets: few studies were found about with fish predators, and data gathered from fishers’ LEK was usually more detailed than literature data (Table 2). Therefore, most of the hypotheses from fishers’ LEK in Ilhabela Island regarding fish predators had medium likelihood according to the criteria of Silvano & Valbo-Jorgensen [15], as data from the biological literature were missing and thus data from fishers’ LEK are the only available information. As above mentioned for fish diet, fishers’ LEK about fish predators is also gathered from direct observations in the environment, such as when fishers inspect their nets and see some organisms eating the entangled fish [41,125]. Fish predation by dolphins, which is locally called boto, has been also mentioned by fishers in other studies of fishers’ LEK in the south, southeastern and northeastern regions of the Brazilian coast [103,105,124]. However, there are few available biological studies about dolphin’s feeding [126], and the information here provided from fishers’ LEK may be thus invaluable to inform future research and conservation not only of fish, but also of dolphins and other large mammals that rely on fish for food.

The interviewed fishers in Ilhabela Island mentioned the sea otter as predator of some fish species (Table 2, Figure 2), but this aquatic mammal has not been mentioned in the biological literature. Interestingly, both coastal fishers in Guaraqueçaba (southern Brazilian coast) and freshwater fishers in the Piracicaba River (southeastern Brazil) also mention otters as being important fish predators in these two aquatic ecosystems [27,41]. Considering that the biology and feeding behavior of sea otters are poorly known in Brazil, this information from fishers’ LEK may be a useful indicator of the potential ecological role of this aquatic predator, as well as of potential conflicts between fishers and this threatened mammal species. In the north Atlantic, a drastic reduction in the population of sea otters due to hunting has caused an increase in the abundance of herbivorous sea urchins, which overgrazed the kelp forests and altered the whole ecosystem [126]. In the northeastern Brazil, freshwater fishers show a conflicting and antagonistic relation with otters, often attacking these mammals when they eat fish from fishers’ nets [44]. Therefore, fishers’ LEK here reported may be useful to evaluate ecological services provided by sea otters and to check for potential conflicts between fishers and these aquatic mammals.

The few scientific studies that address fish predators of the studied fishes usually mention sharks and sea birds (Table 2), but the main predators mentioned by fishers were large reef fishes, such as the caranha (*Lutjanus* spp.) and the mero (*Epinephelus itajara*) (Figure 2). This can be considered as a low likelihood hypothesis, or an overall disagreement between LEK and scientific data, which deserves to be investigated in more detail and can generates new ecological insights [1,15]. In the Caribbean reefs, large groupers (*Epinephelus* spp.) are important fish predators, which can even control invasive exotic fishes [127]. The mero is a large grouper, which was formerly widely distributed along the Brazilian coast but now this fish had its abundance severely reduced by overfishing [5]. Therefore, fishers’ LEK could provide invaluable information about feeding behavior of this rare and threatened fish [5]. Fishers’ LEK here reported in Ilhabela Island indicated that the mero is an important predator of several fish species, and therefore the drastic decrease in the abundance of this large aquatic predator may have caused relevant ecosystem changes [126], which are still poorly known by scientists.

## Conclusions

This study provided a wealth of information about the trophic interactions of 24 coastal fishes in the southeastern Brazilian coast from fishers’ LEK. Furthermore, the information here provided are not restricted to fish, as fishers’ LEK about fish predators indicated useful insights about the diet, functional ecological roles and feeding behavior of some threatened and poorly known fish predators, such as sea otter, dolphin and the mero. These data could be used by managers to improve research and management of fishing resources in Brazil and elsewhere, as most of the studied fishes and their predators have a broad distribution [5]. The approach of likelihood of hypotheses could be useful to indicate the more promising aspects to guide the actions of biologists, policy makers and managers. This study followed a strict and rigorous quantitative methodological approach to interpret and analyze LEK, which increases the reliability of LEK data [6] and makes these data more readily understandable by biologists and more applicable to fisheries management. This study thus indicates the value of fishers’ LEK to improve fisheries research and management, as well as the needy to increase the dialogue and collaboration among managers, biologists and local fishers. If the value of fishers’ LEK is properly recognized, this source of knowledge may provide new biological information to support more effective management measures, which local fishers would understand and accept [1,7,15,34].
